# Impact of a Residency-Run Emergency Medicine Podcast Summary on Learning Retention and Engagement

**DOI:** 10.5811/westjem.52913

**Published:** 2026-08-31

**Authors:** Christopher Reilly, Bobbie Ann Adair White, Katherine Pattee, Hannah Blakely, Reuben Strayer, Sergey Motov, Antonios Likourezos, Rukhsana Hossain, Sarah Kabariti, Jefferson Drapkin

**Affiliations:** * Maimonides Medical Center, Department of Emergency Medicine, Brooklyn, New York; † MGH Institute of Health Professions, Mass General Brigham, Department of Health Professions Education, Boston, Massachusetts

## Abstract

**Introduction:**

Podcasts have become a widely used educational tool in emergency medicine. In this study, a podcast was used as the intervention. This podcast consisted of a brief audio summary of weekly in-person conference content produced by residents and faculty. We assessed whether a resident-led, curriculum-based podcast summary improved knowledge retention and learner engagement compared to a standard written summary format.

**Methods:**

This mixed-methods prospective cohort study conducted at a single urban academic hospital included 54 emergency medicine residents and approximately 66 attending physicians. The study unfolded in three phases: eight weeks of standard written conference summaries (control); followed by eight weeks of written plus podcast conference summaries (intervention); and a four-week post-intervention period with surveys. The primary outcome was knowledge retention, measured by weekly postconference quiz scores comparing the written-summary phase (control) with the podcast phase. Secondary outcomes included learner satisfaction, podcast usage patterns (downloads and self-reported listening), and self-reported impact on clinical application of conference content. Data included quiz scores, survey responses, and podcast analytics. Statistical comparisons of quiz performance were made using paired *t*-tests. Thematic analysis of open-ended responses was performed using Braun and Clarke’s framework.

**Results:**

Mean quiz scores were 73.1% (standard deviation [SD] 6.6) during the written phase and 73.7% (SD 14.4) during the podcast phase with no statistically significant difference in weekly quiz performance between conditions (paired *t*-test, *P* = .92). An exploratory linear trend analysis suggested a steeper week-to-week improvement during the podcast phase, but this post-hoc finding should be interpreted cautiously. Although the podcast did not improve knowledge retention compared with the existing written summary, learners reported high satisfaction, perceived reinforcement of key concepts, and convenient asynchronous access.

**Conclusion:**

In this mixed-methods study, a residency-run podcast summary did not improve quiz-based knowledge retention compared with an existing written conference summary. However, learners reported high satisfaction, perceived reinforcement of key concepts, and convenient asynchronous access.

## INTRODUCTION

Over the past 10 to 15 years, podcasts have become integral to modern medical education, particularly in emergency medicine (EM), with numerous content-related podcasts available.^1^ Surveys of EM residents demonstrate widespread use of educational podcasts as a core component of asynchronous learning and that core content is increasingly delivered through this medium.^2^ Longitudinal analyses of popular podcasts show substantial coverage of core content over time.^3^,^4^ Podcasts are particularly attractive to trainees because they are portable, flexible, and readily integrated into busy clinical schedules, allowing learners to review high-yield material during commutes or between shifts.^5^–^8^ Although audio and visual learning modalities have been widely studied, no consistent superiority has been established; outcomes often depend on learner preference and context.^8^,^9^ Podcasts can serve as a concise modality for summarizing key information and offer flexible, on-demand access that learners can integrate into busy clinical schedules, particularly during residency training.^5^–^7^,^10^ This study evaluates the impact of a resident-led curriculum-based podcast on knowledge retention and learner engagement compared to a written summary format.

Podcasts have demonstrated effectiveness in immediate knowledge retention; however, the existing literature has primarily focused on creation, short-term outcomes, and learner satisfaction.^6^–^8^,^11^,^12^ There is a paucity of research evaluating sustained knowledge retention when an original, curriculum-based podcast, produced within an EM residency program, is compared with a standard written conference summary over multiple weeks. While several studies have explored learning satisfaction and feelings associated with podcasts, an information gap in the literature exists regarding the optimal structure of podcasts, higher level learning outcomes, and their integration into formal curricula.^2^,^7^,^12^ Emergency medicine residents widely use podcasts for off-shift learning, highlighting their significance in medical education.^6^ A national survey of EM residents underscores the prevalence of podcast use, with preferences for shorter durations and reported impacts on clinical practice.^2^ Although podcasts have shown promise in supporting classroom teaching with minimal expenses, the existing literature lacks comprehensive research on their efficacy as teaching tools and best practices in podcast creation.^1^,^7^,^13^

While some case-control studies have explored the impact of podcasts in educational settings, none specifically address learning retention in the context of an original EM residency curriculum-based podcast versus a standard EM residency curriculum. For example, a study on electroencephalography readings after a podcast intervention demonstrated podcasts as noninferior to traditional lectures, with some showing score improvement.^14^ However, the literature review reveals a notable gap in assessing learning retention following the implementation of an original curriculum-based podcast.

## METHODS

This prospective cohort study, conducted at a single-center urban teaching hospital, included 54 EM residents and approximately 66 EM attending physicians at this institution. For several years prior to the study, the residency program had used a locally developed written conference summary compiled weekly by chief residents and faculty. Eligibility criteria for participation in this study included all postgraduate year (PGY) 1–3 EM residents and EM attending physicians at the institution. There were no exclusion criteria. The study focused on an educational intervention using a weekly original audio-format EM curriculum-based conference day summary, compared to the standard written format. Our primary outcome measure was the difference in mean weekly postconference quiz scores between the written summary phase and the podcast summary phase, used to evaluate knowledge retention. We also had several secondary outcomes that included the following: learner satisfaction measured through survey responses assessing participant satisfaction with the podcast format compared to the written summaries; podcast engagement tracked by number of downloads per episode and weekly listening data to assess reach and usage patterns; and self-reported impact on clinical application, assessed via open-ended survey responses where residents and faculty described changes in clinical confidence, behavior, or knowledge application related to podcast use.

The standard residency academic conference day format consists of five hours of dedicated lectures and small groups each week, followed by a structured written email summary that has been used for several years at our institution. This summary is compiled by the residents and includes conferences high-yield “pearls,” key diagnostic and management take-aways, and links to selected resources, and is distributed to residents and attending physicians later that same evening. The intervention involved creating an audio summary in the form of a podcast based on the written summary, in which one or two hosts discussed the same high-yield points in a conversational style, expanded on illustrative cases, and highlighted practical bedside applications. The podcast did not introduce new curricular content but mirrored and elaborated on the written summary to support spaced repetition. Learning retention was assessed through post-conference assessments at each conference day the following week, in comparison to the standard written summary.

According to a national survey of EM residents, the ideal podcast length was reported to be 11–30 minutes.^2^ The podcasts recorded in this study were designed to last 10–20 minutes, featuring one to two speakers discussing material from the prior week for spaced repetition and highlighting conference learning pearls. One speaker remained consistent as the primary or secondary investigator, and the second speaker was a member of the department (attending, fellow, resident, etc). To support implementation, the program purchased a PodBean Tech Inc hosting plan (approximately $14 per month, excluding tax) and two Zoom Video Conferencing Inc ZDM-2 podcast microphones (approximately $100 each). Across the 12 episodes produced during the full project period (including pilot and study episodes), the direct financial cost was approximately $400–$450. Faculty and resident time for planning, recording, and editing was estimated at 1–1.5 hours per episode (10 minutes, 30 to 60 minutes, 30 minutes, respectively), in addition to time for quiz and email summary preparation. These estimates were collected retrospectively to characterize the resource requirements of the intervention.

Approximately four weeks prior to the study’s initiation, all residents received a mixed-methods pre-study survey assessing demographics, study habits, and podcast-related preferences using both quantitative and qualitative items. Surveys were developed by the investigative team, informed by prior literature on podcast use in medical education, and piloted with a small group of residents and faculty for clarity; they were not formally validated instruments. Individual responses were tracked using Research Electronic Data Capture (REDCap) surveys distributed to each participant’s institutional email address. This was a self-controlled study in which each resident functioned as their own control. There were no incentives to participate, and participation was not mandatory. The study unfolded in three parts. Part 1 (eight weeks) represented the control condition, during which only the existing written conference summary was provided. Part 2 (eight weeks) represented the intervention condition, during which the written summary was accompanied by a resident-produced podcast summarizing the same content. Part 3 (four weeks) included continued access to both formats and postintervention surveys. Survey data from residents and attending physicians regarding satisfaction and feedback were collected during this third phase of the study. The timing of this survey distribution was intentionally delayed to mitigate potential bias. Prior to the study’s start, residents were informed that the postconference assessments would have no impact on their academic records, evaluations, or standing within the residency program.

Our primary outcome was knowledge retention, operationalized as weekly quiz performance comparing the written and podcast phases (Kirkpatrick level 2: learning).^15^ Secondary outcomes reflected Kirkpatrick levels 1 and 3 and included learner satisfaction with the podcast, perceived understanding and retention of conference material, and self-reported changes in clinical practice attributed to the intervention.^15^

The study was reviewed by the institutional review board on June 26, 2024 and was determined to be exempt as an educational quality improvement/medical education research project. Participation in quizzes and surveys was voluntary, and completion of these instruments was considered implied consent. All data were de-identified prior to analysis.

### Data Collection Procedures

REDCap surveys were used to collect prestudy survey data on podcast structure and learning preferences. Google Forms were employed for creating and collecting postconference assessments and poststudy surveys. Open-ended questions in surveys also captured qualitative insights on perceived learning and feelings of knowledge retention or improvement. Surveys were distributed via a QR code during the conference. We also assessed the number of plays of each podcast, listener demographics, and listening trends through PodBean (distributed across most platforms such as Apple Podcasts and Spotify).

### Data Analysis

Quiz scores were summarized as means and standard deviations for each phase (written-only vs podcast-only) and compared using paired-samples *t* tests with a significance threshold of *P* < .05 (the eight weekly phase means were paired by study week). Likert-scale survey items were summarized using medians and interquartile ranges, and between-group comparisons of ordinal data were performed with nonparametric tests (eg, Wilcoxon rank-sum). Descriptive statistics were used for podcast usage (downloads, platforms, geography). An unbiased external statistician conducted the analysis to ensure impartiality. Open-ended responses from resident and attending postintervention surveys were analyzed using Braun and Clarke’s six-step reflexive thematic analysis approach.^16^ Two experts (CR, HB) in medical education independently reviewed all responses to generate initial codes. The coding team then met to discuss, refine, and organize codes into candidate themes, iteratively revisiting the data to ensure coherence and representativeness. Discrepancies in coding or theme interpretation were resolved by discussion and consensus. Themes were refined until no new concepts emerged, indicating thematic sufficiency.

## RESULTS

Of the 54 eligible residents, 44 (81%) completed the prestudy survey. Most were PGY 1 or PGY 2 (64%), and 52% identified as female. Prior to the intervention, 91% reported listening to at least one medical podcast per month, and 68% preferred episodes shorter than 20 minutes. Written conference summaries were used “often” or “always” by 57% of respondents, typically for brief review rather than in-depth study. Baseline resident characteristics and pre-study learning preferences are summarized in Table 1.

Weekly quiz scores were collected over eight weeks using a standard written summary and then for eight weeks using the podcast summary. Each weekly quiz consisted of six to eight multiple-choice questions aligned with that week’s conference objectives. Questions were authored by the study team (core EM faculty and senior residents) and reviewed by at least one additional EM faculty member for accuracy and clarity. Items targeted application of key diagnostic and management principles rather than simple recall; although not formally validated, the quizzes followed a consistent blueprinting process to ensure parallel structure across weeks. Quiz participation varied over time, with a median of 38.5 residents per week across the full 16-week study period (39.5 during the written-summary phase and 33.0 during the podcast phase). Participation declined later in the academic year, which may have reflected a combination of off-service rotations that limited conference attendance, quiz fatigue because assessments were voluntary and not tied to formal evaluation, and individual decisions not to participate for unknown reasons.

The mean quiz score during the written summary phase was 73.1% (SD 6.6), while the podcast phase yielded a mean score of 73.7% (SD 14.4). There was no statistically significant difference in weekly quiz performance between phases (paired *t* test, *t* = −.11, *P* = .92), indicating that the primary outcome, knowledge retention as measured by quiz scores, did not improve with the podcast intervention compared to the existing written summary approach. An exploratory linear trend analysis suggested a steeper week-to-week improvement during the podcast phase (+5.3% per week vs +1.2% per week for the written phase), but this post hoc finding should be interpreted cautiously and cannot be taken as definitive evidence of superior effectiveness. Figure illustrates the mean weekly quiz scores during the written (control) and podcast (intervention) phases across the study period.

Table 2 illustrates the mean weekly quiz scores during the written (control) and podcast (intervention) phases across the 16-week period.

Postintervention surveys were collected from both resident and attending physicians. Among residents, 81% completed the prestudy survey vs 48% completing the poststudy survey. Among the 26 residents who completed the postintervention survey, 18/26 (69.2%) reported listening to the podcast at least once a month. Self-reported listening frequency was 6/26 (23.1%) at least weekly, 6/26 (23.1%) every two weeks, and 6/26 (23.1%) once a month; 8/26 (30.8%) reported never listening. Resident and attending postintervention survey responses are summarized in Table 3 and Table 4.

We were unable to track individual-level podcast usage, and platform analytics did not distinguish our residents and faculty from external listeners. As a result, we could not determine the proportion of eligible participants who listened to each episode or correlate individual listening behavior with quiz performance. This substantially limits our ability to attribute quantitative outcomes directly to the podcast intervention. Of those who engaged with the podcast, 60% reported improved retention, 50% felt the podcast improved their understanding of conference material, and 70% were moderately to very satisfied with the format. Residents predominantly engaged with the podcast during commutes or while exercising. Preferences for podcast length aligned with national survey data, with 65% of residents preferring episodes under 20 minutes.^2^

Among attending physicians, 66.7% (8/12) reported listening to at least some episodes of the conference-summary podcast, and 83.3% (10/12) reported being satisfied or very satisfied with the podcast as an educational tool. Attendings commonly described the podcast as a useful mechanism to stay aligned with the residency’s weekly conference themes and to reinforce high-yield clinical concepts. Some attendings reported that topics discussed in the podcast overlapped with clinical cases on shift and perceived that residents were drawing on conference content in bedside discussions. Representative quotations illustrating this perception are presented in the qualitative themes (Table 5). Open-ended survey responses from both residents and attendings were analyzed using Braun and Clarke’s six-step thematic analysis.

During the study period (October 2024–February 2025), the conference-summary podcast episodes were downloaded a total of 413 times across all platforms. Approximately 50% of downloads occurred via Spotify, 26% via Apple Podcasts, and the remainder through other applications or the embedded web player. Based on PodBean analytics, 86% of downloads originated from the United States, with additional downloads/plays from Brazil, Australia, and Hong Kong. Because the hosting platform did not track user identity or institutional affiliation, we were unable to determine what proportion of downloads represented residents, faculty, or external listeners, nor could we reliably confirm whether episodes were listened to within the week after the conference as intended.

## DISCUSSION

This prospective cohort study evaluated a resident-led podcast intervention in a graduate medical education setting, specifically in EM. While our primary outcome—mean quiz scores between podcast and written summary groups—revealed no statistically significant difference, the upward trend in performance observed during the podcast period suggests a potential benefit to learner engagement and knowledge retention. However, as this trend was explored in post hoc analysis, it must be interpreted with caution and cannot be taken as conclusive evidence of effectiveness. Further studies with prespecified trend analyses are warranted. Importantly, the podcast format aligns with key principles of adult learning theory: spaced repetition; autonomy; and relevance to clinical practice.^8^,^9^

The sustained and growing engagement with the podcast episodes was demonstrated by a total of 413 downloads and consistent listening trends in this time frame and reinforces the educational value of this intervention. Because we were unable to track who was specifically downloading the podcasts and where those downloads were happening, we can only weakly infer that most podcast downloads in the United States were by our residents and faculty. In EM, where asynchronous, on-demand learning is particularly critical, our findings highlight how a podcast can function as both a primary and reinforcing mode of content delivery. For many residents, revisiting high-yield conference pearls in an accessible, conversational format promoted spaced repetition, reflection, and confidence in applying knowledge during clinical shifts.

Resident feedback revealed that the podcast encouraged engagement during conference and created a platform where learners felt represented. This may have been especially true when their peers were featured as co-hosts. This could be because of the personal connection felt and the familiarity of hearing peers’ voices. Faculty respondents echoed similar themes, noting that the podcast helped bridge the gap between academic day learning and bedside application. Implementation required only basic recording equipment, institutional support, and a shared vision. This makes this a low-stakes, high-yield intervention adaptable to diverse educational settings.

Qualitative analysis of open-ended survey responses reinforced several key themes in podcast-based learning. First, reinforcement through repetition emerged as a central benefit. Learners described the podcast as a valuable tool for reviewing and reinforcing core content. This finding aligns with prior research demonstrating that podcasts are an effective medium for spaced repetition and retention of key concepts over time.^1^, ^13^, ^17^, ^18^ In particular, students reported that repetition through audio content helped solidify complex material and enhance long-term memory formation.

Second, clinical confidence and application were repeatedly cited by participants. Residents noted that engaging with the podcast helped to improve their real-time clinical decision-making and diagnostic acumen. This perception echoes results from prior studies where podcast-based learning contributed to increased self-confidence in applying medical knowledge in clinical settings.^14^, ^17^, ^18^ It appeared that participants who engaged with podcasts felt more prepared to apply knowledge in practical scenarios, supporting the idea that this format promotes knowledge transfer and clinical relevance.

Third, asynchronous accessibility was frequently identified as a major advantage. Residents appreciated the flexibility of engaging with educational material during commutes or other tasks, which enabled integration of learning into their daily routines. Previous studies suggest that learners favor podcasts because of their portability and accessibility, allowing for just-in-time learning that fits within busy clinical schedules.^5^–^7^ The convenience and mobility of podcasts have been cited as key reasons for their adoption in health professions education, further validating this theme.

Together, these themes support the role of the podcast not only as an educational tool, but also as a vehicle for learner empowerment, reflection, and behavioral change. More broadly, the podcast may have contributed to a cultural shift within the residency program. Even among those who did not listen regularly, the podcast became a recognizable and respected academic product in the department. Its presence reinforced key teaching points, provided structure for longitudinal review, and demonstrated a commitment to educational innovation. This study’s model offers a replicable blueprint for programs seeking to enrich learning retention and asynchronous engagement without adding significant burdens to faculty or learners.

Framed within the Kirkpatrick evaluation model, this study provides relatively strong evidence at level 1 (Reaction): Learners and faculty generally reacted positively to the podcast, found it acceptable, and appreciated its accessibility and format. At level 2 (Learning), our primary outcome showed no difference in objective quiz performance knowledge retention between written summaries alone and written summaries plus podcast, indicating that the intervention did not measurably enhance short-term quiz performance. Perceptions of improved understanding and retention should therefore be interpreted as subjective impressions rather than evidence of superior learning. At level 3 (Behavior), some residents and attendings reported that podcast-derived content influenced on-shift discussions or decision-making; however, these data are self-reported and not supported by direct observation or performance metrics. We therefore regard level 3 findings as exploratory and insufficient to claim a demonstrable behavior change.

This study did not demonstrate an improvement in knowledge retention, our primary outcome, when comparing a resident-run podcast summary with the existing written conference summary. Weekly quiz scores were similar across phases, suggesting that simply adding an audio summary, in the format used here, was not sufficient to achieve a measurable change in performance. First, both conditions provided structured postconference reinforcement, so the written summary itself may already have been an effective tool against which it was difficult for a brief podcast to show incremental benefit. Second, podcast uptake among residents was likely variable and could not be linked to individual quiz performance. Third, episodes were constrained to 10 to 20 minutes and did not include embedded questions or retrieval-practice elements, which may be necessary to translate passive listening into measurable gains. Future interventions could incorporate learner-tracking, interactive features, or targeted support for residents who do not routinely engage with existing written resources to improve the likelihood of detecting a meaningful effect.

## LIMITATIONS

This single-center study may limit generalizability. Listener identity and engagement could not be directly correlated with quiz performance, and the study period may not have captured long-term clinical impact. Initial resident resistance (stemming from perceptions of added responsibility), was mitigated by reframing the podcast as a creative, voluntary contribution. Recruiting consistent faculty contributors also proved challenging due to competing demands and unfamiliarity with podcasting. There was also attrition in resident participation in the poststudy survey. Although 69.2% of resident postintervention respondents reported listening at least once, we could not verify episode-level engagement or link listening behavior to quiz outcomes, and platform analytics could not distinguish resident and faculty downloads from external listeners. Pre- and poststudy survey completion rates differed (81% vs 48% among residents), raising the possibility of response bias, as those more favorable toward the podcast may have been more likely to complete the postintervention survey. We were unable to reliably link individual survey responses, quiz performance, and podcast usage, which limits our ability to correlate engagement with outcomes. In addition, the timing of poststudy survey completion varied, and we did not systematically account for the interval between exposure to specific conference content and survey response, which may have influenced recall. Despite these limitations, the consistent trends in engagement, satisfaction, and perceived learning impact support the feasibility and educational value of residency-run podcast initiatives.

## CONCLUSION

In this mixed-methods, self-controlled study, the addition of a resident-run conference-summary podcast to an existing written summary process did not improve short-term knowledge retention, as measured by weekly postconference quiz scores. Despite this negative primary outcome, learners and faculty reported high levels of satisfaction with the podcast and described it as a convenient, engaging adjunct to the existing curriculum. These findings suggest that, in the context of an already robust written and in-person curriculum, podcast summaries may function more as a learner-preferred adjunct than as a clearly superior modality for improving objective quiz performance.

## Figures and Tables

**Figure:**
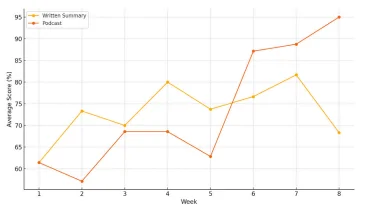
Weekly mean scores during written and podcast phases.

**Table 1:** Baseline resident characteristics and prestudy learning preferences.

Eligible Residents	54
Prestudy survey respondents	44/54 (81.0%)
Most were PGY 1 or PGY 2	64.0%
Female	52.0%
Listened to at least one medical podcast per month before study	91.0%
Preferred podcast episodes under 20 minutes	68.0%
Used written conference summaries often or always	57.0%

* Percentages reflect available pre-study survey data reported in the manuscript

*PGY*, postgraduate.

**Table 2:** Weekly quiz scores by study phase.

Week	Written Summary Mean Score, %	Podcast Summary Mean Score, %
1	61.4	61.4
2	73.3	57.1
3	70.0	68.6
4	80.0	68.6
5	73.8	62.9
6	76.7	87.1
7	81.7	88.8
8	68.3	95.0

**Table 3:** Postintervention survey results for residents (n = 26).

Item (Likert 1–5*)	Residents Responding “Agree” or “Strongly Agree” (≥4), %
Listened to the podcast during commute or exercise	70
Podcast improved understanding of conference material	50
Podcast improved retention of key concepts	60
Felt more confident applying concepts from the podcast on shift	50
Overall satisfaction with the podcast (moderately to very satisfied)	70

* 1 = strongly disagree, 5 = strongly agree.

**Table 4:** Attending postintervention survey responses (n = 12).

Item (Likert 1–5*)	Attending Physicians Responding “Agree” or “Strongly Agree” (≥ 4), %
Listened to the podcast regularly	66.7
Observed residents applying knowledge that overlapped with podcast content on shift	83.3
Podcast improved engagement with residents around conference topics	83.3
Overall satisfaction with the podcast (moderately to very satisfied)	83.3

**Table 5:** Qualitative themes and representative quotations in a study that assessed whether a resident-led, curriculum-based podcast summary improved knowledge retention and learner engagement compared to a standard written summary format.

Theme	Description	Representative Quotations*
Reinforcement through repetition	Podcast episodes helped learners revisit and consolidate key conference “pearls” over time.	“Definitely helps reinforce information.” (Resident) •; “Repetition really helped things stick.” (Resident)
Clinical confidence and application	Learners reported greater confidence and broader differentials when managing patients on shift.	“It makes me more confident in my decisions.” (Resident) • “It helped me expand my differential diagnoses.” “I had a resident on shift who referenced something directly from the podcast—we were able to build on it in real time.” (Attending)
Asynchronous accessibility	The audio format enabled learning during commutes, exercise, or after missed conferences.	“Useful for days I missed conference but still wanted to review.” (Resident) • “Easy to listen to on my commute.”

* Quotations paraphrased/de-identified for brevity and anonymity.
